# Mesenchymal stem cells, autoimmunity and rheumatoid arthritis

**DOI:** 10.1093/qjmed/hcu033

**Published:** 2014-02-10

**Authors:** J.J. El-Jawhari, Y.M. El-Sherbiny, E.A. Jones, D. McGonagle

**Affiliations:** From the ^1^Leeds Institute of Rheumatic and Musculoskeletal Medicine, St. James University Hospital , WTBB, LS9 7TF University of Leeds, UK and ^2^Clinical Pathology Department, Faculty of Medicine, Mansoura University, Mansoura, Egypt

## Abstract

The vast majority of literature pertaining to mesenchymal stem cells (MSC) immunomodulation has focussed on bone marrow-derived MSC that are systemically infused to alleviate inflammatory conditions. Rheumatoid arthritis (RA) is the commonest autoimmune joint disease that has witnessed significant therapeutic advances in the past decade, but remains stubbornly difficult to treat in a subset of cases. Pre-clinical research has demonstrated that bone marrow, adipose, synovial and umbilical cord-derived MSC all suppress the functions of different immune cells thus raising the possibility of new therapies for autoimmune diseases including RA. Indeed, preliminary evidence for MSC efficacy has been reported in some cases of RA and systemic lupus erythromatosis. The potential use of bone marrow-MSC (BM-MSC) for RA therapy is emerging but the use of synovial MSC (S-MSC) to suppress the exaggerated immune response within the inflamed joints remains rudimentary. Synovial fibroblasts that are likely derived from S-MSCs, also give rise to a cell-cultured progeny termed fibroblast-like synoviocytes (FLS), which are key players in the perpetuation of joint inflammation and destruction. A better understanding of the link between these cells and their biology could be a key to developing novel MSC-based strategies for therapy. The review briefly focuses on BM-MSC and gives particular attention to joint niche synovial MSC and FLS with respect to immunoregulatory potential therapy roles.

## Background

The autoimmune diseases are a heterogeneous group of self-directed inflammatory disorders which is characterized by progressive tissue destruction with a loss of function and potentially death if not adequately treated.^1^ Although the pathogenesis of autoimmune diseases are largely played out by cells of the adaptive immune response including B and T cells, one autoimmune disease, rheumatoid arthritis (RA), is also associated with an aberrant joint fibroblast activation that contributes to joint destruction.^2^ The field of mesenchymal stem cells (MSC) research was initially based on harnessing their remarkable multi-lineage differentiation capabilities for skeletal regeneration, including bone and cartilage. Although this remains a major translational focus of regenerative medicine, more recently, another remarkable ability of MSC, namely immunomodulation—has also emerged.^3^ Generally, the immunomodulatory effect of MSC and in particular synovial MSC (S-MSC) has led to introduce these cells as potential therapeutic tools to correct the breakdown of immune tolerance in RA, particularly for a group of cases that are inadequately treated.

## The mechanisms of MSC immunoregulation

The effects of MSC on immune cells have been most extensively studied using ‘gold standard’ bone marrow-MSC (BM-MSC) as the BM being the site of the original discovery of MSC. Basically, BM-MSC could exert widespread modulatory effects on cells of both the innate and adaptive immune responses. Some of MSC effects on T cells include an inhibition of CD4+ T cell proliferation in response to mitogens (e.g. phytohaemagglutinin or concanavalin A) or antibodies (anti-CD2/CD3/CD28).^4–6^ Furthermore, MSC can inhibit the production of IL-2, TNF-α by T cells.^7^ BM-MSC can also induce the differentiation of classic CD4^+^CD25^hi^FOXP3^+^ T regulatory cells (T-regs) and maintain their inhibitory function.^8^^,^^9^ These effects of MSC on T cells have been shown to be dependent on IFN-γ.^4–6^

In addition to the regulation of T cell-mediated immune response, BM-MSC was found to be capable of inhibition of B cell function and differentiation.^10^ Moreover, the chemotaxis of B cells into inflammatory sites could be suppressed via a reduced surface expression of the chemokine receptors; CXCR4, CXCR5 and CCR7 on B cells. These effects on chemokine receptors have been shown when B cells are in co-culture with MSC and are IFN-γ dependent.^10^

In relation to innate immune cells, BM-MSC inhibit the generation of dendritic cells (DCs) from monocytes^11^ and reduce the expression of human leukocyte antigen DR (HLA-DR) and CD80 and CD86 co-stimulatory molecules on antigen presenting cells (APC).^12^ Additionally, the production of the pro-inflammatory cytokines such as IL-2, IFN-γ and TNF-α by APC is reduced and the production of IL-10 is promoted due to the effect of MSC.^12–14^ With respect to NK cells, MSC can reduce the proliferation of both resting and IL-2 activated NK cells, their cytotoxic capabilities and IFN-γ production.^14^

Numerous immunoregulatory mechanisms of MSC have been described including the secretion of Indoleamine 2, 3-dioxygenase (IDO). IDO can catalyse an essential amino acid tryptophan into kynurenine, which impairs the synthesis of various cellular proteins and leads to inhibition of cell proliferation.^15–17^ Other soluble factors produced by MSC include Nitric oxide synthase (iNOS), which induces the production of nitric oxide from macrophages thus inhibiting the proliferation, the secretory and the cytolytic functions of T cells.^18^^,^^19^ Additional immunosuppression mechanisms mediated by MSC involve hepatocyte growth factor, TGF-β, Hemeoxygenase-1 enzyme and HLA-G5.^4^^,^^20^^,^^21^ Besides soluble factors, cell-to-cell contact between MSC and T cells can also suppress the function of T cells that acquire regulatory phenotype marked by a sustained expression of CD69 and increased transcript levels of T-reg related genes.^22^

## Niches and origin of S-MSC

**Figure 1.**
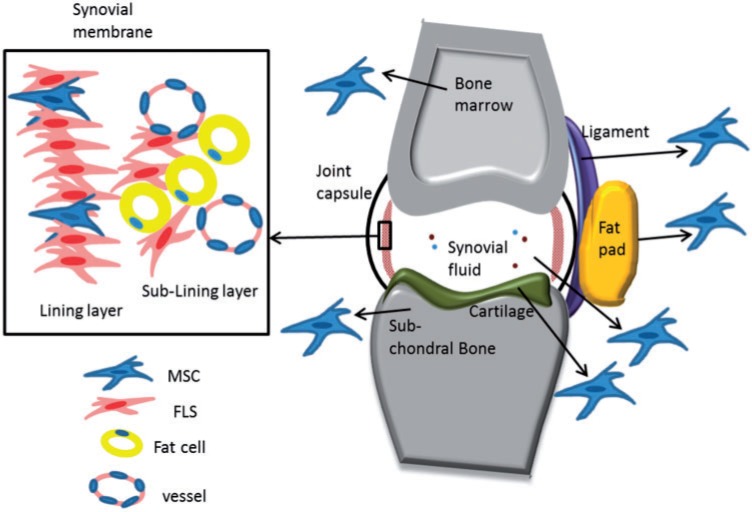
MSC in the joint. Besides BM-MSC, other MSC can be present in multiple areas within the joint; the synovial lining, synovial fluid, joint fat pad, cartilage, subchondral bone and ligaments. Other synovial stromal cells, including FLS and fat cells exist within synovial lining and sublining layers.

It has been shown that MSC can be present in various areas of the joint (Figure 1). The presence of MSC isolated from the joint was first demonstrated in the synovium.^23^ As chondrogenic precursors, MSC are present within the superficial layer of the articular cartilage.^24^ Furthermore, it has been shown that MSC can be derived from certain joint ligaments, menisci and adipose tissue.^25^ Besides tissues, MSC can be detected in the synovial fluid and have been shown to be increased in numbers in some pathological conditions such as osteoarthritis (OA) or following joint injury. However, the quantity of these MSC is negatively related to the degree of synovitis in RA.^26–28^ Initially, MSC were thought to be brought into synovium via migrating blood vessels. This was proposed as MSC are mostly located around blood vessels and because the frequency of MSC is positively correlated with increase tissue vascularity.^29^^,^^30^ However, other studies have demonstrated that S-MSC could be originated within the synovium and exist in projections of the synovial lining compared with sublining layer. Additionally, the morphology and gene expression profile of synovial fluid MSC have been shown to be similar to S-MSC rather than BM-MSC supporting further the synovial origin of these MSC.^27^^,^^28^^,^^31^^,^^32^

## Heterogeneity of synovial stroma: fibroblast-like synoviocytes and S-MSC

The synovial membrane lines the non-articular surface of the joint forming a cavity containing synovial fluid which aids smooth joint locomotion. In health, the synovial membrane is one or two cell thick and formed of two cell types, macrophage-like cells (type A synoviocytes) and stromal cells known as type B synoviocytes. Fibroblast-like synoviocytes (FLS) is the culture-expanded population derived from digested synovial stromal cells, the same population from which culture-expanded S-MSCs are derived.^23^ It is possible that this nomenclature actually represents the same culture-manipulated cell populations. Although culture-expanded BM-MSCs are derived exclusively from the CD271+ population, there is much more complexity with respect to native S-MSCs where a unique phenotype has not thus far been defined.^33^ The main characterization of FLS involves limited proliferation and the surface expression of CD44 and vascular cell adhesion molecule-1 (VCAM-1).^34^ S-MSC-like BM-MSC express classic stromal markers CD105, CD73 and CD90 and lack the expression of haematopoietic markers CD45, CD34, CD14, CD19, CD11b and CD79a.^35^ The colony forming capacity and high levels of proliferation have been used to select S-MSC from the stromal plastic adherent fraction following *in vitro* expansion of synovial digestion.^27^ Although the morphological examination of S-MSC suggests their close relationship to FLS,^36^ these two types of cells seem to be distinctive. A higher level of CD105 and CD166 is detected on S-MSC relative to FLS.^27^^,^^37^ In summary, although they are closely related, no consensus has yet been reached on which markers would permit an isolation of non-overlapping, pure population of S-MSC and FLS.

## Immunomodulatory capacity of healthy S-MSC and FLS

**Figure 2.**
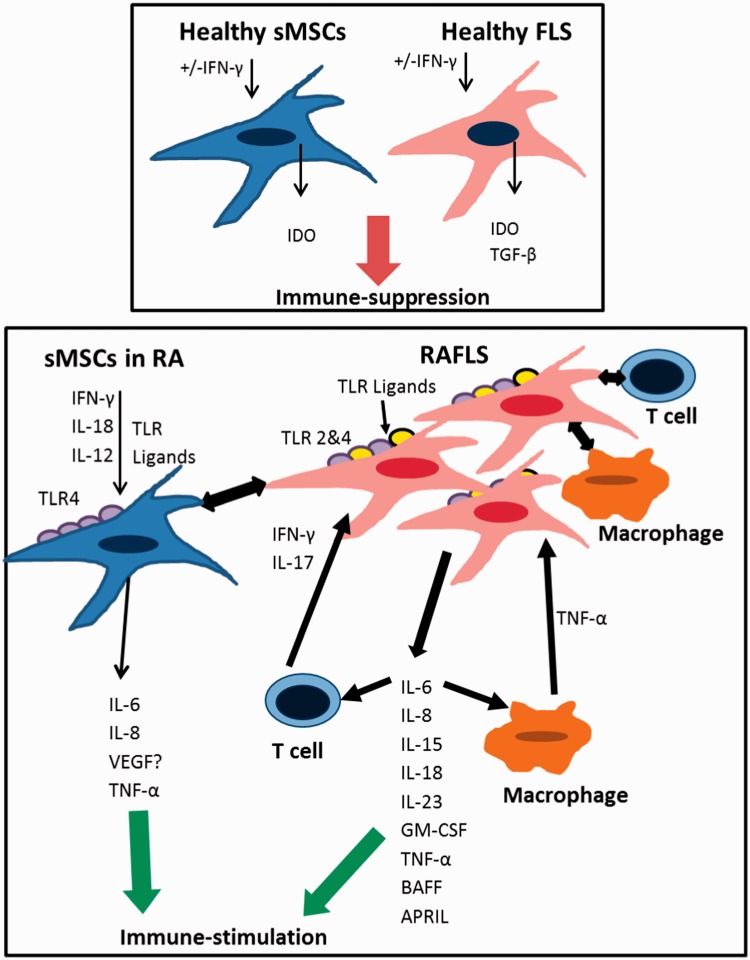
Synovial stromal cells in health and RA. (**A**) In healthy condition, both FLS and MSC can perform immunosuppressive effect via secretion of IDO and/or TGF-β. (**B**) In RA, S-MSC could be activated by RA FLS and/or by IFN-γ, IL-18 and IL-12 to produce pro-inflammatory cytokines IL-6, IL-8 and TNF-α. Furthermore, RA FLS are activated by T cells or macrophages by cell-to-cell contact or by the effect of TNF-α, IL-17 or IFN-γ. RA FLS in turn produce pro-inflammatory cytokines, IL-6, IL-8, IL-15, IL-18, IL-23, GM-CSF, TNF-α, BAFF and APRIL, which all cause immune stimulation.

In contrast to BM-MSC, the immunoregulatory role of healthy S-MSC is less documented. S-MSC extracted from healthy subjects are able to inhibit T cell proliferation.^38^^,^^39^ Similar to BM-MSC, S-MSC extracted from OA patients can maintain the percentage of T regs when in co-culture with T-reg enriched lymphocytes from healthy donors.^40^ However, the effect of S-MSC on B cells, NK cells or APCs remains to be explored. One documented mechanism by which S-MSC can display their suppression effect on T cells is via the production of IDO^39^ (Figure 2, A). It has been shown that S-MSC and BM-MSC can display similar IDO activity at a basal level or when induced by IFN-γ and TNF-α.^39^ Therefore, a similar inhibitory effect by S-MSC could be expected on other immune cells.

In physiological conditions, It is plausible that healthy FLS can also be involved in immune haemostasis within the joint (Figure 2, A). It has been shown that FLS could inhibit T cell proliferation in a similar manner to fibroblasts derived from other tissues such as skin, gingiva and cornea.^16^^,^^41–43^ Healthy FLS could also inhibit the differentiation of monocytes into DC via IL-6 dependent mechanism.^44^ Human fibroblasts are generally known to mediate their immunoregulatory role via IDO-dependent mechanism and TGF-β which FLS can produce.^44^^,^^45^

The immunoregulatory function of MSC can be modulated by the pro-inflammatory cytokines such as IFN-γ (the key factor), TNF-α, IL-1α or β.^46^ This phenomenon has been recently termed the MSC ‘licensing’ and the production of soluble IDO, iNOS and PGE2 by MSC has been found to be enhanced by licensed MSC.^47^^,^^48^ The process of licensing of MSC is also critically linked to the activation of Toll-like receptors (TLRs) expressed on the MSC surface. A differential polarization of MSC towards either inflammatory or anti-inflammatory side has recently shown to be related to type of TLR primed. Stimulation of TLR4 causes the production of pro-inflammatory cytokines IL-6, IL-8 or TGF-β generating MSC1 phenotype. On the contrary, binding of specific ligands to TLR3 induces immunosuppressive MSC2 cells which produce IDO.^49–51^

Like MSC, various types of fibroblasts have been shown to be ‘licensed’ by IFN-γ.^41^^,^^43^ Therefore, it can be assumed that FLS can be also activated by IFN-γ to display immunoregulatory effects. As IFN-γ has a central role in the licensing process, joint inflammatory milieu could be critical for the stromal control of the local immune response.^52^ It is possible that the synovial stromal cells, in all stages of maturation: as progenitors (S-MSC) or as mature cells (FLS), control the ‘healthy’ balance of immune response within the healthy joint and the failure of this haemostasis probably takes place with an excess inflammation.

## S-MSC and FLS in RA

It has been shown that S-MSC harvested from RA is capable of immunosuppression *in vitro.*^39^ However, it seems that RA micro-environment including inflammatory cells and cytokines causes an inefficacy of S-MSC to control the exaggerated immune response in this disease (Figure 2, B). One mechanism that could explain ineffective control of the immune response by S-MSC in RA is TLR activation which is directly linked to RA pathogenesis.^53^ TLR2 and 4 are hardly detected in healthy synovium compared with RA synovium where these TLRs are highly expressed particularly in the synovial lining layer.^54^ The most abundant types of ligands for TLR receptors that detected in RA synovium are those binding to TLR2 and TLR4 which when activated could induce the release of pro-inflammatory cytokines by macrophages.^53^ Examples of these ligands include HSP22^55^ and the extracellular matrix component Biglycan.^56^ Interestingly, the expression of Biglycan has been detected in the lysate of RA FLS^57^ indicating that RA FLS can be involved in inappropriate priming of S-MSC. RA-induced cytokines, IL-12 and IL-18 together with IFN-γ, can cause an upregulation of TLR4 on MSC and thus trigger the expression of IL-6 and TNF-α.^54^^,^^58^^,^^59^ Altogether, this suggests that in RA, S-MSC could be induced via inflammatory cytokines to express higher level of TLR4 and consequently respond to different TLR4 stimulants. Additionally, this indicates that the process of licensing and mechanism of immunosuppression displayed by S-MSC could be reversed in favour of RA progression.

RA FLS have been described as activated and ‘aggressive’ cells that directly aggravate the inflammatory processes (Figure 2, B).^60^ RA FLS have been shown to acquire MHC class II compared to healthy FLS and work as antigen presenting cells leading to T cell activation and proliferation in a comparable way to APCs.^61^^,^^62^ Despite that RA FLS lack the surface expression of classic co-stimulatory molecules such as CD80, FLS-dependent T cell proliferation is related to an interaction between CD47 on T cell surface and its ligand, thrombospondin-1 expressed by FLS.^63^ Also, RA FLS induce the activation and accumulation of T cells following an interaction between CXCR4 on T cells and its ligand stromal cell-derived factor-1 (SDF-1) on RA FLS.^64^

Similarly, RA FLS enhance B cell recruitment, survival and functions, particularly via SDF-1 and VCAM-1-dependent mechanisms.^65^^,^^66^ RA FLS also promote the survival of B cells via an increase of the expression of BCL-XL by B cells^67^ and by upregulation of IL-15 receptor on the surface of B cells.^68^ RA FLS induce immunoglobulin class switching in B cells via production of high levels of TNF ligand family member, B-cell activating factor (BAFF) and a proliferation-inducing ligand (APRIL).^69^ With respect to innate cell functions, RA FLS can act as APCs and could express high level of surface Toll receptors TLR 2 and 4.^61^^,^^70^ Priming by cytokines such as IL-1 or TNF-α also induces the production of GM-CSF from FLS which is a major macrophage activating factor.^71^ In conclusion, this all demonstrates that RA FLS can promote both types of immune responses.

There is an indication as shown *in vitro*, that synovial stromal cells can be immunosuppressive.^38^ The aggressive pro-inflammatory nature of RA FLS shown *in vivo* could be explained by the effect of RA micro-environment and interaction between RA FLS and immune cells. FLS express intracellular molecules such as Cryopyrin which are inducible by TNF-α and can in turn increase the production of IL-6 and IL-8.^72^ Also, type-II collagen-specific effector T cells could stimulate secretion of IL-15, IL-18 and TNF by FLS which in turn activate the production of IFN-γ by T cells.^73^ In summary, polarization of RA FLS into pro-inflammatory cells appears to be related to RA environmental factors and this seems to create a vicious circle leading to chronicity of this disease.

The development of RA is associated with hyperproliferation of FLS, a process which could be related to the inhibition of FLS apoptosis.^74^^,^^75^ Additionally, increased production of cysteine-rich protein 61 (Cyr61), a molecule involved in cell adhesion and migration, stimulates FLS proliferation.^76^ The factors driving FLS proliferation in RA do not appear to be acting on S-MSC; one study has shown that the frequency of S-MSC in the RA synovium is reduced compared with OA controls and this is correlated with the progression of synovitis.^27^ This imbalance in the numbers of aggressive RA FLS and MSC could be another contributing factor leading to the loss of immune haemostasis in RA.

In summary, RAFLS and S-MSC could directly participate in the progression of inflammation in RA by different means (Figure 2, B). Despite their potential immunosuppressive role, these synovial-derived stromal cells are not effective in controlling inflammation in RA and potentially in other auto-inflammatory diseases. More knowledge on the immunoregulatory function of S-MSC in health and early stages of RA could therefore lead to new methods of targeting inflammatory processes in the RA synovium.

## S-MSC as cell therapy for RA—principles

The use of autologous MSC for immunosuppression therapy is widely accepted because these MSC can efficiently inhibit the proliferation of activated lymphocytes in a similar manner to allogeneic healthy cells as *in vitro* studies have shown.^77^ Although the morphology and phenotype of cultivated S-MSC is similar to that of BM-MSC, it has been shown that S-MSC has superiority in several aspects. Interestingly, S-MSC have much greater proliferative rate compared to bone marrow as well as muscle- and fat-derived MSC as shown in animal models.^78^ Furthermore, S-MSC retains their proliferative power regardless of donor age and after several passages in culture unlike other MSC.^23^^,^^79^ Comparing S-MSC with skin-derived MSC has shown that the later exert less immune suppressive properties than S-MSC, particularly upon stimulation with IFN-γ.^39^ In summary, S-MSC could be a convenient MSC source to consider for RA cell therapy.

The extraction of S-MSC from pathological joints such as OA and RA is usually performed as part of surgical treatment such as the removal of degenerated cartilage.^80^ An extraction of small quantity of synovial tissue is usually sufficient to extract MSC effectively.^23^ MSC can be extracted from different sites within a joint; suprapatellar pouch, infrapatellar fat pad and medial outer or medial inner articular regions. However, the functional capabilities of S-MSC could be varied from other joint-driven MSC. It has been noticed that MSC harvested from medial outer tissues have higher proliferation rate compared with those extracted from other joint regions.^30^ This point reflects a need for more research on the differences between immunosuppressive capacities of MSC derived from various anatomical areas within the joint.

In animal models of RA, allogeneic MSC have been shown to exhibit poor immunogenicity *in vitro.*^81^^,^^82^ Furthermore, animal model of collagen-induced arthritis (CIA) responded successfully to allogeneic BM-MSC delivered intraperitoneally.^83^ Despite the apparent advantages of allogeneic MSC, some conflicting data have emerged in terms of RA treatment. Allogeneic MSC extracted from BM, umbilical cord and placental cultures were rejected in a mouse model of graft versus host disease, did not form ectopic bone and lost their immunosuppressive power that was displayed *in vitro.*^84^^,^^85^ However, studies performed on patients of autoimmune diseases have demonstrated the advantage of using allogeneic MSC. The Using of autologous MSC did not seem to improve SLE disease course despite an increase of proportion of peripheral blood T-regs.^86^ MSC extracted from SLE patients grew slower in culture, display less viability and produce less TGF-β in contrast to allogeneic healthy MSC indicating that the former cells are probably defective in function.^87^ In case of RA, it has been shown that intravenous infusion of allogeneic BM-MSC or Umbilical Cord (UC)-MSC into small group of anti-TNF resistant cases induces a temporary clinical improvement but not on long term follow-up.^88^ A recent work has proved the safety and the potential efficiency of allogeneic MSC for RA therapy in a larger number of patients.^89^ Furthermore, the combination of allogeneic UC-MSC and conventional therapy improves RA cases clinically and serologically. The same research group has conducted a pilot study for 15 patients with refractory SLE and has shown a clinical improvement and stability in renal function after treatment with allogeneic BM-MSC.^90^ In summary, using allogeneic MSC could be effective in refractory autoimmune disorders such as RA and SLE; however, more clinical trials are needed to approve these findings.

The best tool to deliver MSC is still undetermined. Although MSC can migrate into the specific site of inflammation, MSC infused systemically could display anti-inflammatory responses in different sites which underpin an effective use of MSC in multi-organ diseases.^91^^,^^92^ However, the systemic administration of autologous MSC could cause an exacerbation of the disease as shown in CIA model although the mechanism is not clear.^93^ Furthermore, there is a potential activation of hidden or low grade tumours because MSC could suppress the anti-tumour immune response.^94^ Consequently, there is now an increasing trend to implement a local MSC delivery, such as intra-articular MSC injections. A recent study using SCID mouse model of arthritis has verified the practicability and safety of using intra-articular adipose-derived MSC injection as a therapy of rheumatic disorders.^95^ Similar assessment is needed to test if a direct inoculation into joint could be the convenient way of the S-MSC delivery in RA cases.

## How S-MSC can be an effective tool for therapy of RA; Conclusions and future aspects

Within the normal synovial tissue, two types of non-haematopoietic stromal cells: FLS and S-MSC appear to play an important role in controlling the inflammation and immune haemostasis. In normal conditions, closely related FLS and S-MSC can act as immunomodulatory cells controlling the magnitude of immune responses. Both stromal lineage cells retain some level of immunosuppressive capability during pathological conditions such as RA, which can be detected *in vitro*. However, due to various factors within RA milieu and as a result of a direct contact with inflammatory cells and cytokines, the immunomodulatory function of S-MSC and FLS seem to be disturbed. The proliferation of RA FLS which acquire aggressive pro-inflammatory phenotype within the synovium takes the upper hand. Moreover, the function of S-MSC seems to be shifted towards immune stimulation. Based on these considerations, the use of S-MSC as cell therapy for autoimmune disorder such as RA needs to be complemented with targeting the inflammatory factors within the synovium. Essentially, further investigation into the interaction between S-MSC and RA FLS and with immune cells is still required to improve the therapy of RA. Some pre-clinical studies have been shown that targeting of signalling molecules in active pro-inflammatory cells including RA FLS could be of value in treatment of RA.^96^ Therefore, combination of MSC therapy together with targeting RA FLS must be considered. Furthermore, joint MSC harvesting sites, doses as well as routes and schedules of delivery remain underexplored and merit further investigation before this type of therapy could become a clinical reality.

## Funding

This work was partially funded through WELMEC, a Centre of Excellence in Medical Engineering funded by the Wellcome Trust and EPSRC (grant no. WT 088908/Z/09/Z).

*Conflict of interest:* None declared.
